# A systematic proximity ligation approach to studying protein‐substrate specificity identifies the substrate spectrum of the Ssh1 translocon

**DOI:** 10.15252/embj.2022113385

**Published:** 2023-04-19

**Authors:** Nir Cohen, Naama Aviram, Maya Schuldiner

**Affiliations:** ^1^ Department of Molecular Genetics Weizmann Institute of Science Rehovot Israel

**Keywords:** BirA, proximity labeling, Sec61, Ssh1, translocation, Methods & Resources, Organelles

## Abstract

Many cellular functions are carried out by protein pairs or families, providing robustness alongside functional diversity. For such processes, it remains a challenge to map the degree of specificity versus promiscuity. Protein–protein interactions (PPIs) can be used to inform on these matters as they highlight cellular locals, regulation and, in cases where proteins affect other proteins ‐ substrate range. However, methods to systematically study transient PPIs are underutilized. In this study, we create a novel approach to systematically compare stable or transient PPIs between two yeast proteins. Our approach, Cel‐lctiv (CELlular biotin‐Ligation for Capturing Transient Interactions *in vivo*), uses high‐throughput pairwise proximity biotin ligation for comparing PPIs systematically and *in vivo*. As a proof of concept, we studied the homologous translocation pores Sec61 and Ssh1. We show how Cel‐lctiv can uncover the unique substrate range for each translocon allowing us to pinpoint a specificity determinator driving interaction preference. More generally, this demonstrates how Cel‐lctiv can provide direct information on substrate specificity even for highly homologous proteins.

## Graphical abstract

Capturing transient interactions via a cellular biotinylation approach, Cel‐lctiv, allows for direct, proteome‐wide substrate range comparison between two homologous yeast translocation pores.

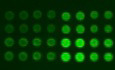

## Introduction

A major driver of the evolution of cellular functions is duplication of genes followed by specialization of the homologous pair (Fenech *et al*, 2020). While gene duplication and specialization broaden functionality, provide cellular robustness and increase regulatory capacity, they pose major challenges when trying to uncover the specialized function of each homologous protein product. The difficulty is increased by the fact that despite exhibiting some specificity, that enables diversification of their functions, they also often demonstrate some degree of functional overlap—thus providing backup for each other (Ihmels *et al*, 2007). Obviously, this becomes even more challenging as the number of homologs increases and large protein families are formed. Hence, there is a general need in cell biology for approaches able to describe the particular range of any given protein's functions *in vivo*, even when that protein belongs to a large and closely related family. For proteins that act on other proteins, such as kinases, proteases, chaperones, and more, these approaches would entail uncovering their exact substrate range as a means to deciphering their specificity or promiscuity.

Methods to study the selectivity of proteins with similar functions often utilize *in vitro* approaches, and this requires observing a single (or very few), well‐behaved, substrates. *In vivo* approaches can also be used, and these usually ablate or alter one protein and assay the cellular outcome of this manipulation. However, when one protein is silenced or eliminated, substrates may reroute to backup pathways (giving rise to false negatives). Alternatively, compensatory rewiring of the cellular system may cause indirect effects (giving rise to false positives). Thus, to unravel substrate range of homologous proteins and reveal the basic principles driving their specialization, there is a clear need for new approaches. These should rely on systematic techniques that would work well for many types of homologs. Such methods should also have the capacity to work *in vivo* and provide information on cellular activity preference without deleting the tested protein itself.

One powerful way to uncover the specialized cellular functions that differentiate homologous proteins is by assaying their stable, and transient, interactions. Knowing these interactions may suggest different cellular contexts in which these proteins work (e.g., different cellular locals or different complex members), unique regulatory mechanisms (such as various post‐translational modification enzymes working on them) and, for the subgroup of proteins working on other proteins as substrates, also their unique and specific substrate range.

In this study, we utilize the yeast, *Saccharomyces cerevisiae* (from hereon called simply yeast) to create a novel, systematic, approach to measure the differential stable and transient interactomes for proteins that govern parallel functions as a means to map their functional specificity. Our approach relies on a well‐established biotinylation platform—the specific BirA biotin ligase and its acceptor peptide AVI tag (Beckett *et al*, 1999). On this platform, we establish a methodology for high‐throughput pairwise proximity biotin ligation assays for uncovering protein–protein interactions systematically and *in vivo*. We have named it Cel‐lctiv (pronounced like “selective”—CELlular biotin‐Ligation for Capturing Transient Interactions *in vivo*). Cel‐lctiv overcomes the limitation of hypothesis‐driven, small number of candidates by assaying the entire proteome and the false positive/negative issues around loss of function by maintaining the complete cellular context.

As a proof of concept, we chose to use the two homologous translocation pores into the endoplasmic reticulum (ER)—together catering for a huge fraction of the proteome that must be translocated into the secretory pathway. The well‐studied translocation pore is the Sec61 translocon (Bieker & Silhavy, 1990; Deshaies *et al*, 1991), while the less studied homolog is Ssh1 (Sec Sixty‐one Homolog 1) (Finke *et al*, 1996). Since translocation through a pore is an extremely transient event and since Sec61 and Ssh1 have overlapping functions, they have therefore provided a challenge for uncovering the repertoire of translocating substrates in the past. Since the presence of a Sec61 homolog is conserved from yeast to humans (The human SEC61A1 homolog is termed SEC61A2), it stands to reason that there is an evolutionary purpose or advantage for maintaining the pair; however, this has not yet been uncovered.

In the past, attempts to uncover the reason for having two homologous translocons have focused on hypothesis‐driven, single candidates and measured their translocation capacity either *in vivo* or *in vitro*. These classic studies have been extremely important in providing the first candidate substrates for Ssh1 (Wittke *et al*, 2002; Spiller & Stirling, 2011; Aviram *et al*, 2016). However, from this handful of potential candidates, each having distinct properties and no clear unifying aspect, it has been difficult to determine the rules governing specificity. Beyond the small number of potential proteins tested to date, an additional complication has been the use of loss of function approaches. Since Sec61 is an essential protein but Ssh1 is not (albeit its loss renders the cells extremely slow growing; Wilkinson *et al*, 2001), most such experiments were done unidirectionally on cells deleted for Ssh1. This has led to the misconception that Ssh1 is simply a backup translocon with potentially only a small number of unique substrates none of which are essential under normal growth conditions.

By combining Cel‐lctiv to assay the interaction preference of both Ssh1 and Sec61 coupled with whole proteome analysis on the effects of *ssh1* loss, we uncover the range of cellular effects of losing Ssh1 as well the degree of functional overlap between Sec61 and Ssh1 and their unique potential substrate range. Moreover, having a wide range of new substrates enriched for each translocon allowed us to decipher a biochemical specificity determinator for their interaction preference. More generally, this demonstrates how Cel‐lctiv can provide direct information on substrate selectivity and specificity even for highly homologous proteins and does this in the native cellular environment on minimally perturbed proteins and in a systematic fashion.

## Results

### Development of Cel‐lctiv—cellular biotin‐Ligation for capturing transient interactions *in vivo*

**Figure 1:**
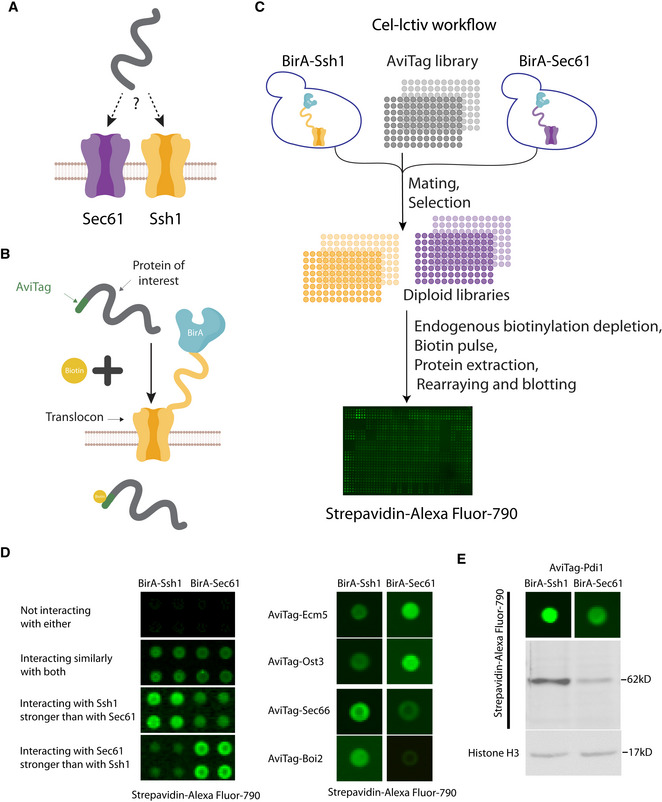
Cel‐lctiv—CELlular biotin‐Ligation for Capturing Transient Interactions *in vivo*—is a powerful approach for measuring protein specificity AA schematic illustration of the research question—how can we define differential substrate range for two homologous proteins? Shown as an example are the two endoplasmic reticulum translocons Sec61 and Ssh1.BA schematic illustration of the Cel‐lctiv approach to uncover the repertoire of stable and transient protein–protein interactions. When a protein (in this case one of the translocons) is tagged with the specific biotin ligase BirA and the potential substrate is tagged with the biotin acceptor, AviTag, even a transient physical interaction will allow biotinylation of the AviTag and this can be read out using fluorescent streptavidin.CA schematic illustration of the work flow of Cel‐lctiv: Strains containing the BirA tagged proteins (in this case either one of the two translocons) were mated with a collection of ~ 5,330 yeast strains in each of which one protein is N‐terminally tagged with AviTag under the native promoter and localization signal. Following this procedure, some strains were lost and the final strain number was 4,812. Each resulting diploid strain underwent automated protein extraction, blotting onto a membrane and visualization using fluorescent streptavidin. Biotinylation levels were compared between the two samples to identify an interaction preference.DCel‐lctiv results were clustered into groups: proteins that did not show a detectable interaction with either translocon, proteins that showed similar interaction with both translocons, and proteins that showed a preference for one of the translocons. Examples of the latter groups are shown (*n* = 9).EComparison between the dot blot and western blot analysis of the same lysate from the screen to verify that the signal difference results from differential substrate biotinylation (*n* = 9 for the dot blot and *n* = 3 for the western blot).

To uncover the different cellular environment, regulatory pathways, and substrates of a protein machinery, it is essential to capture not only their stable interactions but also the very transient ones. For example, when trying to understand the distinct roles of the two translocons Sec61 and Ssh1, it would be important to understand which proteins are transiently translocating through them in an *in vivo* setting (Fig 1A). While assaying transient interactions between two proteins is feasible (Villalobos *et al*, 2007), it was for long difficult to do this systematically on a proteome‐wide level in yeast. We have recently set up novel approaches in yeast to harness the power of nonspecific biotinylating ligases such as BioID2 (Roux *et al*, 2012) and TurboID (Branon *et al*, 2018) to uncover transient interactors (Fenech *et al*, 2023). Despite the method being robust, when trying it on Sec61 and Ssh1 that should cater for hundreds of substrates, we only observed a handful of highly expressed proteins for each. Our interpretation of this is that since all biotinylation of TurboID strains occurs in parallel there is competition from the highly abundant and strong interactors and this does not leave a possibility to detect the low‐abundance and more transient interactions that we would like to measure. Hence, we developed a new approach for systematically comparing both stable and transient PPIs between two proteins in a pairwise manner that we call Cel‐lctiv (CELlular biotin‐Ligation for Capturing Transient Interactions *in vivo*).

Cel‐lctiv relies on proximity labeling of the specific biotin ligase, BirA, to its unique acceptor peptide AviTag (Beckett *et al*, 1999). However, instead of doing this for a single pair of proteins, Cel‐lctiv measures the pairwise interaction abundance for a given protein with every other protein in the yeast proteome. The benefit of using such an approach is that each interaction event results in a separately measured output so the sum of interactions can be quantified to provide a global analysis of biotinylation levels per protein pair. Comparing the same AviTagged substrate with different BirA tagged proteins (e.g., two homologs) allows the direct comparison of their relative interaction preference (Fig 1B). Unlike the nonspecific biotin ligation methods (Roux *et al*, 2012; Branon *et al*, 2018), there is only a single, identical, ligation site for each protein enabling a linear relationship between the number of molecules that have interacted and the signal intensity. This property is especially important in proteins that are co‐translationally translocated where only a single opportunity for biotinylation is available and where only a small portion of the protein N terminus is available for labeling. Hence, despite the fact that BirA enzymatic activity is slower than both BioID2 and TurboID (Branon *et al*, 2018), since our assay is performed in a pairwise manner and we measure the relative biotinylation level of two strains, we are able to assay quantitatively the differential in labeling even of low‐abundance proteins that could be masked in pooled approaches. This is especially important when trying to uncover a complete substrate range.

One major difficulty with using the pairwise biotinylation as a robust tool, especially if relying on endogenous expression levels as we have done (see below), is the high background of endogenous biotinylation in yeast. To reduce the background biotinylation, we have therefore utilized our previously established methodology for reducing endogenous biotinylation, ABOLISH (Auxin‐induced BiOtin LIgase diminishing; Fenech *et al*, 2023). This allowed us to reduce background noise and increase our sensitivity, especially for low‐abundance proteins.

To develop Cel‐lctiv, we utilized two recently established yeast strain collections (libraries). In one library, each protein in the yeast proteome is tagged with an N‐terminal AviTag and expressed under its endogenous promoter and localization signal (such as signal peptide [SP] or mitochondrial targeting signal [MTS]) if it exists. In the second library, each protein is similarly tagged with an N‐terminal BirA retaining the natural promoter and targeting information (Fenech *et al*, 2023). To perform Cel‐lctiv, a protein pair or family of interest are selected from the BirA library (we selected BirA‐Sec61/Ssh1). Then, the BirA strains are mated with the whole‐genome AviTag collection that is of the opposite mating type. This generates new diploid whole‐genome collections carrying both selected traits. In our case, each contains one of the translocons (Ssh1 or Sec61) tagged with BirA and all yeast proteins tagged with AviTag (Fig 1C).

To enable the quantitation of thousands of pairwise interactions in quadruplicate, we developed a high‐throughput dot blot approach using fluorescent streptavidin to visualize the degree of biotinylation in each strain following the background biotinylation reduction by ABOLISH (Fig 1C). The results are then computationally analyzed to measure relative biotinylation signal for each BirA and AviTag combination.

Focusing on our protein pair, Sec61 and Ssh1, both of which were tagged with BirA such that it was facing the cytosol, we found proteins that did not interact with either, that interacted with both and, using a cutoff of a twofold higher signal, those that favor a specific translocon over the other. Specific interactors include Emc5 and Ost3 interacting preferentially with Sec61 while Sec66 and Boi2 interacted preferentially with Ssh1 (Fig 1D). To quality control our method, we remeasured a select set of protein extracts from the original assay by resolving them on a SDS–PAGE gel, thus allowing us to assay the specific AviTag biotinylation contribution to the total biotinylation and ensuring proper molecular weight as expected from that strain (Fig 1E).

Collating the complete set of proteins (Datasets EV1 and EV2), we found that both previously suggested substrate of Ssh1 that we could assay, Sec66 (Spiller & Stirling, 2011) and Prc1 (Wilkinson *et al*, 2001), indeed showed a preference for Ssh1 supporting the ability of Cel‐lctiv to uncover the transient interactome of two homologous proteins.

### Cel‐lctiv provides a global insight into Ssh1 substrate selectivity

Looking globally at the whole proteome, we found that, as expected, the majority of proteins did not interact with either translocon (2,742, note that in this group we cannot differentiate between biological and technical reasons). From the interacting proteins, many had similar signal levels for both translocons, suggesting that they are either nonspecific interactions or that there exists a high degree of functional overlap (1,674). Importantly, our Cel‐lctiv approach uncovered many proteins that have a preference for one translocon.

There are many different reasons why proteins may be biotinylated by Sec61 or Ssh1. Biotinylated proteins may be complex members or they may be soluble cytosolic components that are regulators or we may have false positives that only happen to be in proximity such as highly abundant cytosolic proteins. Only a fraction of our interactions are expected to be true substrates. However, for one group of proteins it is clear that they must be substrates and these are signal peptide (SP) containing proteins. SP containing proteins are often soluble, residing inside the lumen of the ER or later secretory pathway organelles, therefore only enabling their interaction with the cytosolically facing BirA during translocation. In cases where SP‐containing proteins also harbor a transmembrane domain (TMD), their most likely topology would be with their tagged N′ facing the lumen of the ER (so‐called Type I proteins). The only way such proteins could be biotinylated by the slow BirA on the cytosolic surface is if they passed through the pore of the translocon.

**Figure 2:**
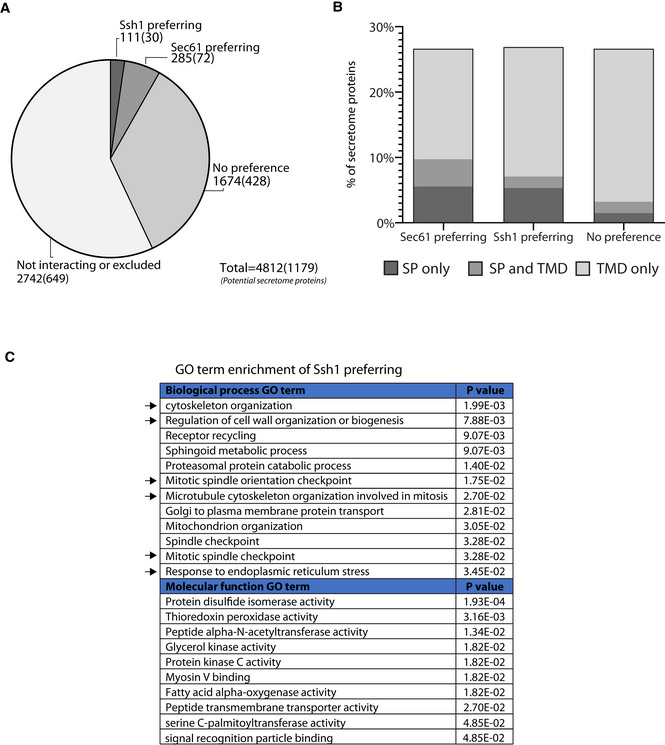
Cel‐lctiv provides a global insight into Ssh1 substrate selectivity AA pie chart showing the distribution of Cel‐lctiv results divided into noninteractors/excluded, equally interacting with both or differential preference proteins. Numbers represent all proteins that fit into the group with those in parentheses referring to the subgroup that could be direct substrates (predicted to have a SP or at least one TMD and no MTS).BA bar graph showing the abundance of different features within the set of secretory proteins that came up in the various interaction groups.CGO term enrichment analysis of proteins that showed an interaction preference with Ssh1, using Yeastmine. Black arrows indicate terms that intersect with the GO term enrichment analysis of *type="InMathematical_Operators">∆ssh1*‐affected proteins (Fig 4).

Focusing therefore only on those proteins that have a SP a TMD or both (but removing those that have a predicted MTS, since those could be mitochondrial inner membrane proteins), we found that they constitute 30 out of the 111 proteins that showed a preference for Ssh1. Two hundred and eighty‐five proteins showed a preference for Sec61, with 72 of them potential secretory substrates (Fig 2A; Datasets EV1 and EV2). This already suggests that while Ssh1 and Sec61 can indeed act as backups for each other for the vast majority of potential substrate proteins, they also each have a unique function since tens of proteins preferred to translocate specifically through one, or the other, under the ploidy and growth condition that we measured. Interestingly, the abundance of soluble secretory proteins containing only a SP without a TMD was higher in the group of proteins that showed an interaction preference to one of the translocons when compared to the group of proteins that showed no preference (Fig 2B). This supports recent evidence that the pore‐forming translocons may be most important for soluble secretory cargo (Smalinskaitė *et al*, 2022).

Amidst the biotinylated proteins, we also found differential interactions with potential regulators such as the kinases Gin4, Pkc1, Ptk2, and Kdx1 interacting with Ssh1 in comparison with Bck1 and Iks1 interacting with Sec61. These differential kinase‐translocon interactions suggest that they are either affected by or affect different regulatory networks (Datasets EV1 and EV2). Surprisingly, 12.6% of the interacting proteins of Ssh1 were from organelles that are thought to be separate from the secretory system, such as mitochondria (Dataset EV1).

To assay for cellular processes that are overrepresented in the group preferentially interacting with Ssh1, we turned to Gene Ontology (GO) term enrichment analysis (Cherry *et al*, 2012; Engel *et al*, 2014; Fig 2C; Dataset EV3). The analysis brought up the expected GO terms that describe the core translocation machinery such as “Signal recognition particle binding” and “peptide transmembrane transporter activity.” However, some unexpected GO terms also came up as enriched highlighting processes related to cytoskeleton organization, cell wall biogenesis, and response to ER stress (Fig 2C). Altogether these suggest that there is a functional divergence of the specific substrates that utilize Ssh1.

### A high‐throughput screen uncovers the global effect of *Δssh1* on the proteome

**Figure 3:**
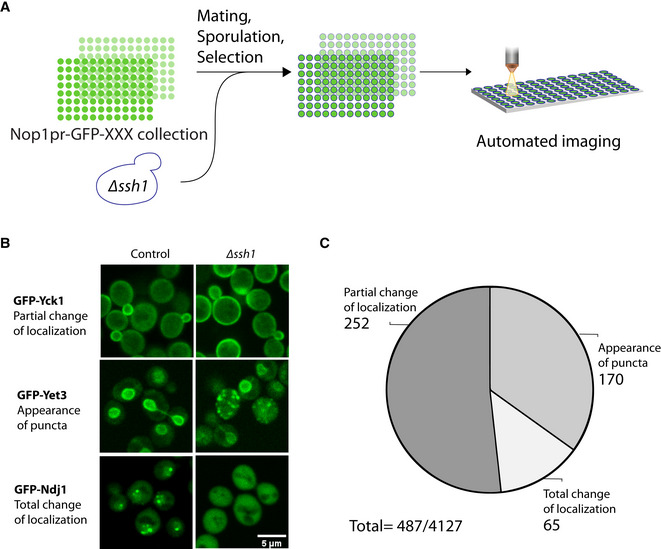
High‐throughput screen uncovers the effect of *Δssh1* on protein localization AA schematic illustration of the high‐throughput screen aimed to identify the cellular effects of losing Ssh1. A strain where *ssh1* was deleted was integrated into a library of ~ 5,500 yeast strains in each of which one protein is N‐terminally tagged with GFP under the constitutive *NOP1* promotor. Strains were automatically imaged using a high‐throughput spinning disk confocal microscope and manually examined to uncover proteins that show a change in signal shape and/or localization.BRepresentative strains from the three groups into which all hits were divided: Proteins demonstrating a partial change in localization, proteins that showed an appearance of puncta, and proteins that showed a total change in localization. Scale bar = 5 μm.CPie chart showing the overall results from the high‐throughput screen divided into the three categories from B (for the complete list of hits see Dataset EV1).

The Cel‐lctiv approach enabled us to find potential translocating substrates that prefer one translocon over another. However, this gives little insight as to what the physiological outcome of this preference is. One way to assess the physiological effects of having nonoverlapping functions for the translocons is to gauge the cellular effects of losing one of them. To uncover the global cellular effects, those that cannot be buffered by the presence of a homolog, we focused on the nonessential translocon Ssh1. To do so, we visualized all yeast proteins (tagged on their N terminus with a green fluorescent protein [GFP] under the *NOP1* promoter; Yofe *et al*, 2016; Weill *et al*, 2018), on the background of either a *∆ssh1* or a control using a high‐throughput confocal spinning disk imaging platform (Fig 3A). We manually analyzed the images and categorized proteins as affected if the protein changed signal localization and/or shape in the mutant relative to the control. We found 252 proteins that changed their localization (as exemplified by GFP‐Yck1; Fig 3B), 170 proteins that displayed a specific type of partial change of localization with the appearance of punctate structures (as exemplified by GFP‐Yet3; Fig 3B) and 65 proteins that displayed a complete change in their localization (as exemplified by GFP‐Ndj1; Fig 3B; for an overview of all changes see Fig 3C and Dataset EV1). Altogether over 10% of the assayed proteome was affected by Ssh1 loss suggesting a significant and global effect of its loss as well as rewiring of the cell to accommodate the loss of this translocation channel. This highlights its unique cellular role and its nonredundant functions.

### Transient interactors that showed a preference for Ssh1 suggest the mechanistic link to its cellular effects

**Figure 4:**
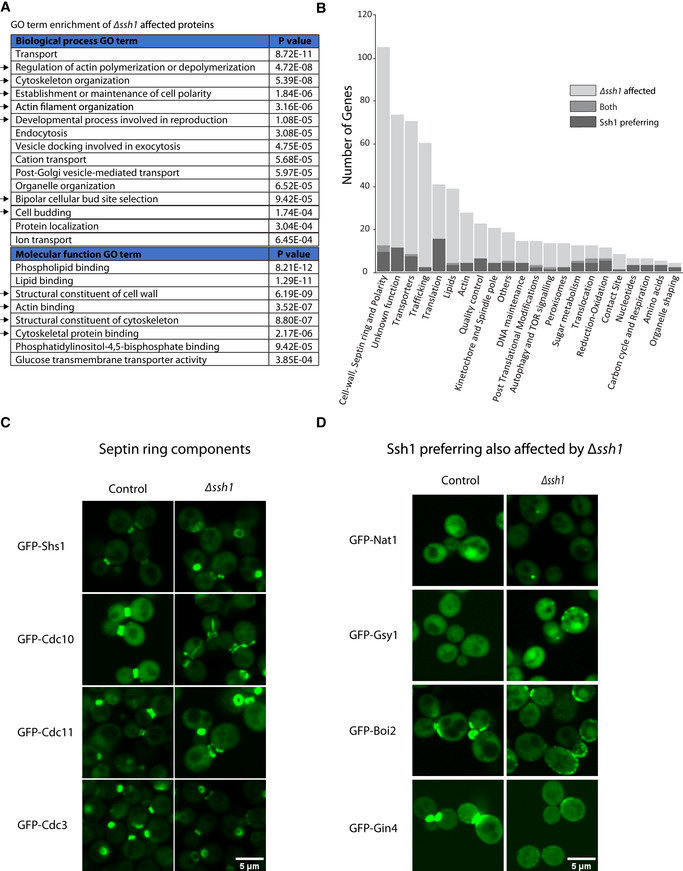
Loss of Ssh1 leads to defects in Septin ring morphology AGO term enrichment analysis of all proteins affect by the *∆ssh1* background. Black arrows indicate terms that intersect with the GO term enrichment analysis of proteins that showed an interaction preference with Ssh1 (Fig 3).BA bar graph of the manually assigned functional groups showing crosstalk between the two screens.CMicroscopy images of N‐terminal GFP‐tagged Septin ring components in the *∆ssh1* and control backgrounds. The comparison shows accumulation of open ring forms and alterations in Septin ring structure in the mutant. Scale bar = 5 μm.DAn example of proteins that came up in both screens showing both an interaction preference for Ssh1 and a localization effect in the *∆ssh1* background. Scale bar = 5 μm.

While the above results show global changes, we wished to uncover the major cellular processes that were affected by the loss of Ssh1. To assay for cellular processes that are overrepresented in the proteins whose localization was altered by the loss of Ssh1, we turned again to GO term enrichment analysis (Cherry *et al*, 2012; Engel *et al*, 2014). The analysis showed enrichment for a variety of processes related to transport and cytoskeleton organization. However, the most consistent and repeating GO term group was those related to budding and polarity process. These include “Establishment or maintenance of cell polarity”; “Development process involved in reproduction”; “Bipolar cellular bud site selection”; “Cell budding”; and “Structural constituent of cell wall” (Fig 4A; Dataset EV3).

Since GO terms are limited in their functional information, and to be able to compare the outputs of the two systematic screens (uncovering proteins that preferentially bind Ssh1 and those that are affected by its loss), we manually grouped the protein hits from the two screens into functional categories based on the description and annotation in the yeast genetics database (SGD) (Cherry *et al*, 2012; Engel *et al*, 2014; Dataset EV1). These were then used to group the hits from the two approaches and visualize those aspects of cellular function that were most represented from both approaches together. Our analysis uncovered that the most enriched groups were “Cell wall, Septin ring and polarity”; “Transporters”; “Trafficking”; and “Translation.” The largest group of proteins represented in both screens were in the “Cell wall, Septin ring and polarity” and, as expected, in “Translocation” (Fig 4B; Dataset EV1).

The major structure that represents the outcome of many of the above cellular processes (cytoskeleton, polarity, and budding) is the Septin ring. The Septin ring is a protein scaffold that assembles on the dividing septum (bud neck) during cell division and is involved in the selection of the bud site, the positioning of the mitotic spindle, polarized growth, and cytokinesis (Longtine & Bi, 2003; Oh & Bi, 2011). The Septin structure matures throughout the cell cycle: It first appears as a distinct ring; after bud emergence, the ring broadens to assume the shape of an hourglass around the mother‐bud neck; finally during cytokinesis the Septin cortex splits into a double ring which eventually disappears. The clear visual structure of the Septin ring prompted us to follow the major Septin ring components in *∆ssh1* strains. Indeed, we found that for all major Septin subunits that we assayed (Shs1, Cdc10, Cdc11, and Cdc3) there was a clear enrichment for the open, ring, stage, and the ring was also enlarged relative to control cells (Fig 4C). This may be an indication of delayed cytokinesis, which would also explain the reduced growth rate of *∆ssh1* strains (Wilkinson *et al*, 2001). Such a delay may be due to the activation of the ER surveillance stress response (ERSU) (Piña & Niwa, 2015).

Indeed, of the 12 proteins that came up in both screens were Boi2 and Gin4, which are directly related to the budding process. Gsy1, a glycogen synthase whose expression was shown to be induced by osmotic shock (Unnikrishnan *et al*, 2003), and the Nat1 N‐acetyltransferase (Fig 4D). Together they suggest a mechanistic link between direct Ssh1 interaction and effects on cell wall. This suggested that specific direct interactors of Ssh1 can explain the physiological effects of its absence on Septin ring formation.

### The biochemical properties of the signal peptide determine translocon preference

**Figure 5:**
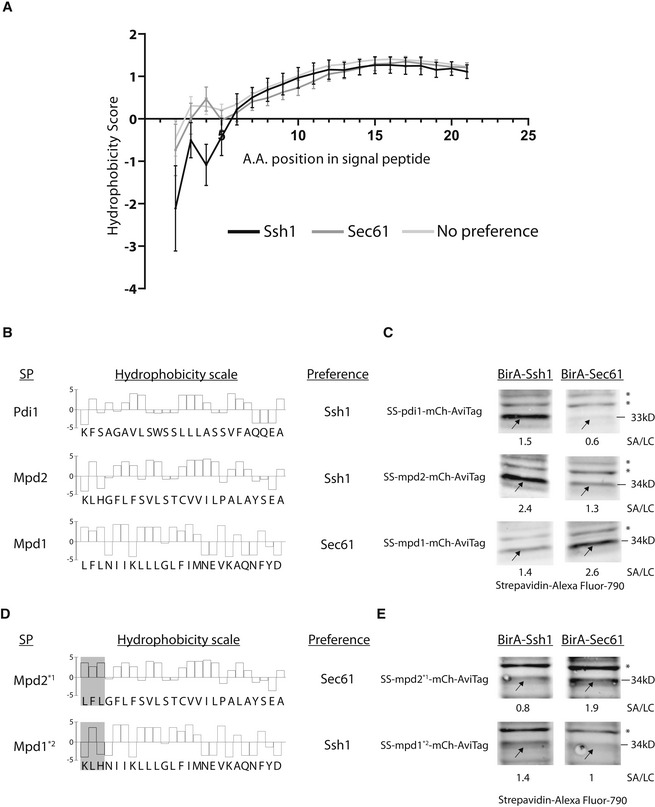
Biochemical properties of the signal peptide determine translocon preference AA graph showing the Kyte–Doolittle hydrophobicity scale for each amino acid average for all predicted SPs from the group of proteins that showed an interaction preference to either Ssh1, Sec61, or that interacted with both similarly the error bars represent the standard error of means of the Kyte–Doolittle hydrophobicity scale (mean ± S.E.M. *n* = 9).BA bar graph showing the Kyte–Doolittle hydrophobicity scale for the predicted SPs of PDI family members that came up as differential interactors in our Cel‐lctiv assay. Shown are Pdi1 and Mpd2 (two PDI family members that showed an interaction preference to Ssh1) and Mpd1 (a protein that showed an interaction preference for Sec61).CWestern blots showing the biotinylation level of the SP‐only reporter construct on a BirA‐Ssh1 or BirA‐Sec61 background. These highlight that the SP is sufficient to endow interaction preference in the absence of the complete protein context. The Streptavidin (SA)/ Loading Control (LC) ratio represents the ratio between the biotinylation signal (band marked by an arrow) and the H3 loading control band. Asterisks (*) mark nonspecific bands (*n* = 3).DA bar graph showing the Kyte–Doolittle hydrophobicity scale for the two variants of Mpd1 and Mpd2 where the three first amino acids were swapped aiming to alter the interaction preference.EWestern blots showing the biotinylation level of the two variants of Mpd1 and Mpd2 (with 3 amino acids swapped in their SP reporter construct) on a Ssh1‐BirA or Sec61‐BirA background. Swapping the first three amino acids is sufficient to convert the SP reporter construct interaction preference. This suggest that the information for translocon choice is found at the very N terminus of the proteins. The SA/LC ratio represents the biotinylation signal (band marked by an arrow) and the H3 loading control band total signal ratios. Asterisks (*) mark nonspecific bands (*n* = 3).

If proteins that have a translocation preference exist, then there should be sequence determinants that encourage translocon choice. Having a list of potential substrates that prefer interacting with (and therefore potentially translocating through), one translocon or another allowed us to explore the biochemical properties that differentiate Ssh1 and Sec61 substrates. We focused on those interactors that contain a SP due to its role in translocon engagement and since those should be enriched for true substrates. For these substrates, we explored the biochemical properties of the SP by comparing the hydrophobicity (using a Kyte–Doolittle scale) across the various positions in the first few amino acids of proteins that prefer either translocon or have no clear interaction preference. We found that Ssh1 interactors have distinct hydrophobicity in their most N‐terminal residues (Fig 5A).

To look at this in more detail, we chose to study three proteins that belong to the Protein Disulfide Isomerases (PDI) family. The PDI family contains five proteins (Nørgaard *et al*, 2001) three of which we could detect in our assay. Surprisingly, all three, Pdi1, Mpd2, and Mpd1 showed an interaction preference for one of the translocons. Pdi1 and Mpd2 showed preference for Ssh1, while Mpd1 showed a preference to Sec61 (Datasets EV1 and EV2). Indeed, looking into their SPs they each had hydrophobicity patterns that suited the preference shown by the group analysis (Fig 5B). Two PDI family members (Eps1 and Eug1) were not represented in the screen at all; however, their SP pattern suggests that they too are Ssh1 substrates.

To assay whether the observed SP properties are sufficient to drive the interaction preference, we generated a construct that contains only the SP of either Pdi1, Mpd1, and Mpd2 fused to a reporter containing an mCherry and an AviTag. By assaying the interaction preference on the background of either BirA‐Ssh1 or BirA‐Sec61, we could observe that indeed the SP alone was sufficient to generate an interaction preference similar to that observed by the full protein (Fig 5C). Hence, small differences in SP biochemical properties underly translocon selectivity.

To specifically demonstrate that the information that lies in the SP is found in the first amino acids, we performed a domain swap experiment in which we exchanged the first three amino acids of the Mpd1 and Mpd2 SP (creating the constructs Mpd1*2 and Mpd2*1; Fig 5D). Indeed, simply exchanging these amino acids was sufficient to alter the translocon preference of a protein (Fig 5E).

Our approach shows how Cel‐lctiv can provide a global view of transient interactions on a global scale. It also demonstrates how having the broad knowledge of potential substrate range enables mining the specific sequence determinants that govern selectivity. Moreover, while the variability of the SP was previously shown to affect targeting pathway choice and the capacity to open the translocon channel (Johnson *et al*, 2013), this is the first demonstration that information in the SP also dictates the choice of translocons and by that, potentially the downstream fate of secretory proteins by different sets of accessory proteins, modifications, and trafficking.

## Discussion

Uncovering the specificity of protein homologs is a challenging task (Herzig *et al*, 2012; Yifrach *et al*, 2016; Megyeri *et al*, 2019). In this manuscript, we have brought forward a new approach for exploring the substrate range for molecular machineries that have proteins as their substrates. Our approach, Cel‐lctiv, relies on proximity ligation in a pairwise setting that enables systematic identification of transient interactions, expected for a protein substrate, even for low‐abundance proteins.

We focused on an enigmatic pair—the two homologous SEC translocons into the ER—Sec61 and Ssh1. Using both systematic loss of function studies to gauge the effect of Ssh1 loss on all cellular proteins as well as discerning the differential interactors of Sec61 and Ssh1, we could not only uncover the repertoire of cellular processes affected specifically by Ssh1 loss but also validate the biochemical properties of the SP as one determinant enabling differential substrate selection.

Why would some proteins evolve to use Ssh1 and others Sec61? An intriguing concept that came out of our analysis is that large protein families may have evolved to distribute their entry between the two translocons to increase robustness of their function. One such example is the PDI family of proteins—out of five members of this family the three that we could measure had distinct preference for either one of the two translocons. Loss of targeting for two of the PDI family members that prefer Ssh1 may explain why its loss leads to higher sensitivity to the reducing agent Dithiothreitol (DTT) (Rand & Grant, 2006) as well as to the glycosylation inhibitor tunicamycin (Kapitzky *et al*, 2010).

An additional hypothesis as to why cells have evolved two entry routes into the ER is that Ssh1 and Sec61 reside in different subdomains of the ER membrane endowing their substrates with a unique post‐translational environment in which to mature. Another reason may be the capacity to regulate differential entry upon changes in cellular conditions—this is hinted upon by the differential set of regulatory proteins that each translocon interacts with. However, to directly test this hypothesis, our Cel‐lctiv approach would have to be performed under additional metabolic or stress conditions. Either way, it is clear that Ssh1 is not simply a backup translocon and has evolved a unique substrate range and regulatory network.

Surprisingly, our analysis uncovers a huge enrichment for cell wall biogenesis and integrity components as both being affected by the loss of *ssh1* and directly interacting with Ssh1. The direct interactions suggest that maybe specific cell wall proteins prefer to utilize Ssh1 as their primary translocon. This includes the Chitin Synthase Csh2 and the putative chitinase Cts2. Another aspect is the potential regulatory role of Ssh1 by binding, anchoring, or modulating regulatory factors such as the bud growth and polarity inducing kinases Gin4, Kdx1, and Pkc1 or phosphatase Msg5 and the Boi1/2 polarity factors required for polarized vesicle fusion.

Another unexpected finding is the presence of mitochondrial membrane proteins as specific interactors of Ssh1. One such protein, Tim21 is a mitochondrial inner membrane protein that both interacts with Ssh1 and is affected by its loss. Tim21 is a typical substrate of the ER‐SURF pathway in which mitochondrial inner membrane proteins utilize the ER surface to target to mitochondria (Hansen *et al*, 2018; Koch *et al*, 2021). Indeed, strains deleted for *SSH1* have previously been shown to have respiratory effects, enhanced mitochondrial DNA loss (Wilkinson *et al*, 2001) as well as altered mitochondrial biogenesis (Laborenz *et al*, 2019).

More generally, our work shows that Cel‐lctiv can be used for the study of protein–protein interactions on a whole proteome level and is especially suited for uncovering short, transient, and weak interactions in a native environment. This method, available to all (all libraries and protocols are distributed freely) and not requiring sophisticated machinery such as mass spectrometers, is especially powerful for uncovering the substrate range of proteins that work on other proteins. In this respect, Cel‐lctiv can enable the dissection of substrate range and specificity of protein pairs, protein families or even just groups of proteins performing parallel functions. These include kinases/ phosphatases; other post‐translational modification enzymes (ubiquitin ligases, glycosyl transferases, etc.); proteases and many more. By uncovering the entire repertoire of differentially interacting substrates, it should be possible to start dissecting the rules governing specificity and promiscuity of proteins, previously difficult to study.

## Materials and Methods

### Yeast strains and plasmids

*Saccharomyces cerevisiae* strains were based on the laboratory strain BY4741 (Baker Brachmann *et al*, 1998). Genetic manipulations were performed using the lithium acetate, polyethylene glycol, single‐stranded DNA method (Gietz & Woods, 2006). Plasmids for PCR‐mediated homologous recombination were previously described (Longtine *et al*, 1998; Janke *et al*, 2004). Dataset EV4 lists the primers, plasmids, and strains used in this study.

### Western blots

#### Culturing and protein extraction

Five milliliter of cells at 0.5OD_600_ was collected by centrifugation at 3,000 *g* for 3 min, washed with 1 ml of double‐distilled water (DDW), resuspended in 200 μl lysis buffer containing 8 M urea, 50 mM Tris, pH 7.5, and oComplete Protease Inhibitors (Merck), and lysed by high‐speed bead beater with glass beads (Scientific Industries) at 4°C for 10 min. 25 μl of 20% Sodium Dodecyl Sulfate (SDS) was added before incubation at 45°C for 15 min. The bottom of the microcentrifuge tubes was then pierced, loaded into 5 ml tubes, and centrifuged at 4,000 *g* for 10 min to separate the lysate from the glass beads. The flow‐through collected in the 5 ml tubes was transferred to a fresh 1.5 ml microcentrifuge tube and centrifuged at 20,000 *g* for 5 min. The supernatant was collected, and 4× SDS‐free sample buffer (0.25 M Tris, pH 6.8, 15% glycerol, and 16% Orange G containing 100 mM DTT) was added to the lysates, which were incubated at 45°C for 15 min.

#### Resolving, blotting, and acquisition

Protein samples were separated by SDS–PAGE using a 4–20% gradient gel (Bio‐Rad) and then transferred onto a 0.45 μm nitrocellulose membrane (Pall Corporation) using a Trans‐Blot Turbo transfer system (Bio‐Rad). Membranes were blocked in 2% wt/vol Bovine Serum Albumin (BSA) in phosphate‐buffered saline (PBS) solution for 30 min at room temperature (RT), incubated for 1 h at RT with rabbit anti‐Histone H3 (ab1791, 1:5,000; Abcam) diluted in a 2% wt/vol BSA/PBS solution containing 0.01% NaN3. After washing, membranes were then probed with secondary goat anti‐rabbit‐IRDye680RD antibody (ab216777; Abcam) and streptavidin‐Alexa Fluor790 (S11378; Invitrogen), both diluted 1:10,000 in 2% wt/vol BSA/PBS solution for 1 h at RT. Blots were washed and imaged on the LI‐COR Odyssey Infrared Scanner. Images were quantified using GelAnalyzer 19.1 (Lazar & Lazar, n.d.).

### High‐throughput microscopy screening

#### Library preparation

The synthetic genetic array (SGA) method was used for integrating the desired genomic manipulations into yeast libraries (Tong & Boone, 2006; Cohen & Schuldiner, 2011). Query strains for screens were constructed on a SGA‐ready strain (YMS721; Breslow *et al*, 2008), and libraries were handled using a RoToR bench‐top colony arrayer (Singer Instruments). Briefly, query strains were mated with strains from the library on rich medium plates to generate diploid cells. Cells were then transferred to nitrogen starvation media for 1 week to induce sporulation. Haploid cells were selected using canavanine and thialysine (Sigma‐Aldrich) lacking leucine to select for MATalpha. The final library was generated by selecting for the combination of manipulations desired. Representative strains from the final library were validated by both microscopy and check‐PCR.

#### Culturing and microscopy

Cells were moved from agar plates into liquid 384‐well plates using the RoToR bench‐top colony arrayer (Singer Instruments). Liquid cultures were grown overnight in synthetic medium with 2% glucose (SD) in a shaking incubator (LiCONiC Instruments) at 30°C. A Tecan freedom EVO liquid handler (Tecan), which is connected to the incubator, was used to back‐dilute the strains to ∼ 0.25 OD_600_ in plates containing the same medium. Plates were then transferred back to the incubator and were allowed to grow for 4 h at 30°C to reach logarithmic growth phase. The liquid handler was then used to transfer strains into glass‐bottom 384‐well microscope plates (Brooks Bioscience) coated with Concanavalin A (Sigma‐Aldrich) to allow cell adhesion. Wells were washed twice in a low fluorescence synthetic medium (Formedium) to remove floating cells and reach a cell monolayer. Plates were then transferred into the automated microscopy system using a KiNEDx robotic arm (Peak Robotics).

Imaging was performed using an automated Olympus SpinSR system using a Hamamatsu flash Orca 4.0 camera and a CSUW1‐T2SSR SD Yokogawa spinning disk unit with a 50 μm pinhole disk. Images were acquired using a 60× air lens NA 0.9 (Olympus), 100 mW 488 nm OBIS LX laser system (Coherent), GFP Filter set [EX470/40, EM525/50] (Chroma).

Images were manually inspected using Fiji‐ImageJ software (Schindelin *et al*, 2012).

### High‐throughput proximity biotin ligation assay

#### Culturing, induction, and protein extraction

Cells were moved from agar plates into liquid 384‐well plates in three biological replicates using a RoToR bench‐top colony arrayer (Singer Instruments). Liquid cultures were grown overnight in low biotin 0.512 nM d‐biotin (sigma) SD medium in a shaking incubator (LiCONiC Instruments) at 30°C. A Tecan freedom EVO liquid handler (Tecan), which is connected to the incubator, was used to back‐dilute the strains to ∼ 0.25 OD_600_ in plates containing no biotin SD media supplemented with 1 mM Auxin (sigma). Plates were then transferred back to the incubator and were allowed to grow for 4 h at 30°C to reach logarithmic growth phase The liquid handler was then used to prepare samples to measure OD using a plate reader (Tecan) and to add high biotin media 30 nM d‐biotin(sigma) to the samples which were then incubated at RT for 60 min. Then, the plates were centrifuged at 3,000 *g* for 3 min using a robotic centrifuge (Hettich), the media were removed, and the cells were resuspended with 25 μl of lysis buffer containing 0.1 M NaOH, 0.05 M EDTA, 2% SDS, 100 mM DTT, 1 mM PMSF (sigma), orange G dye (adopted form von der Haar, 2007) and moved to 384 wells PCR plate (Eppendorf) with reusable lid (4titude), the PCR plates were moved to a robotic thermal cycler (Inheco) and the samples were incubated at 90°C for 10 min, neutralized using 5 μl of 0.5 M acetic acid, and incubated again at 90°C for 10 min.

#### Blotting, development, and image acquisition

The lysate was blotted to a nitrocellulose membrane (pall) using the RoToR into a 1,536 dot array using the 384 long pads (Singer Instruments) calibrated to move 200 pl of liquid in three technical replicates. The membrane was dried overnight at RT and blocked for 30 min with 2% (W/V) BSA in PBS (Sigma), incubated while shaking ON at 4°C with rabbit anti‐Histone H3 (Abcam) antibody used as a control, washed, developed using goat anti‐rabbit‐IRDye680RD antibody (ab216777; Abcam), and streptavidin‐Alexa Fluor790 (S11378; Invitrogen), on an odyssey LI‐COR imaging system.

### Computational analysis

#### Dot blot analysis

Image analysis was performed using a version of Fiji‐ImageJ (Schindelin *et al*, 2012) plugin Protein Array Analyzer (G. Carpentier) modified to retrieve the region of interest (ROI) for each dot in the array. A set of MATLAB scripts were used to measure the signal for each channel for each dot, normalize the signal using the loading control and the three biological repeats and compare the normalized signal for each set of lysates. Altogether we had nine replicates for each set (three biological repeats and three technical repeats). A set was labeled as excluded in the following cases and in cases where the normalized signal showed more than a *Z* score of 10 standard deviations lower than the rest of the membrane and in cases where one of the strains composing the set was missing to begin with as indicated by the pre‐lysis OD measurement. In no strain did we have repeats showing a standard deviation higher than 3 between the measurements indicating the reproducibility of our approach (Appendix Fig S1). A protein was designated as having an interaction preference if a signal with a fold change greater than two was found.

#### SP hydrophobicity analysis

Hydrophobicity analysis was performed using the Kyte–Doolittle hydrophobicity scale MATLAB function (Kyte & Doolittle, 1982).

#### GO term analysis

GO term analysis was performed using Yeastmine (Cherry *et al*, 2012; Engel *et al*, 2014) GO term enrichment analysis with Holm–Bonferroni correction. For the microscopy screen, the background set was defined as all genes included in the SWAT collection (Yofe *et al*, 2016; Weill *et al*, 2018), and for the interaction screen, the background was defined as all genes included in the AviTag library (Fenech *et al*, 2023) and that were not excluded as part of the analysis.

## Author contributions

**Nir Cohen:** Conceptualization; data curation; software; formal analysis; validation; investigation; visualization; methodology; writing – original draft; writing – review and editing. **Naama Aviram:** Conceptualization; supervision; methodology; writing – review and editing. **Maya Schuldiner:** Conceptualization; supervision; funding acquisition; writing – original draft; project administration; writing – review and editing.

## Disclosure and competing interests statement

Maya Schuldiner is an EBM Member. This has no bearing on the editorial consideration of this article for publication. The authors declare no other conflict of interest.

## Supporting information



Appendix S1

Dataset EV1

Dataset EV2

Dataset EV3

Dataset EV4

## Data Availability

The datasets and code used in this study are available at:
Datasets: https://doi.org/10.5281/zenodo.7669532. Code: https://github.com/Maya‐Schuldiner‐lab/Cel‐lctiv.
