# Supplementary material for: A systematic proximity ligation approach to studying protein‐substrate specificity identifies the substrate spectrum of the Ssh1 translocon
Source: EMBO J. 2023 Apr 19;42(11):e113385. doi: 10.15252/embj.2022113385 (PMC10233368; doi:10.15252/embj.2022113385)
Supplement: Supplementary file 1 — Appendix S1 [file EMBJ-42-e113385-s001.pdf]

Table of content

Appendix Figures and Figures legends

Appendix Figure S1: Reproducibility of the Cel-lctiv method 1

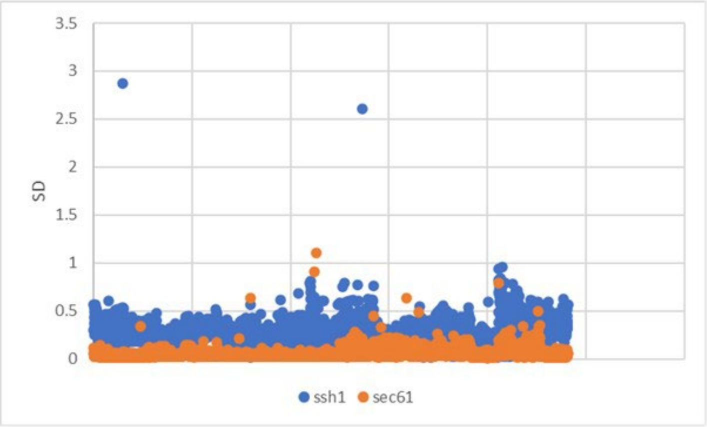

Appendix Figure S1: Reproducibility of the Cel-lctiv method

Plot showing the SD for each set of repeats of the Cel-lctiv method. Shown are 9 repeats in total (3 biological and for each 3 technical). The low SD demonstrates the reproducibility of the Cel-lctiv method for each BirA/AviTag combination
