# Supplementary material for: A systematic proximity ligation approach to studying protein‐substrate specificity identifies the substrate spectrum of the Ssh1 translocon
Source: EMBO J. 2023 Apr 19;42(11):e113385. doi: 10.15252/embj.2022113385 (PMC10233368; doi:10.15252/embj.2022113385)
Supplement: Supplementary file 6 [file EMBJ-42-e113385-s003.pdf]

THE EMBO JOURNAL

A systematic proximity ligation approach to studying protein-substrate specificity identifies the substrate spectrum of the Ssh1 translocon

Nir Cohen, Naama Aviram, and Maya Schuldiner
DOI: 10.15252/embj.2022113385

Corresponding author(s): Maya Schuldiner (maya.schuldiner@weizmann.ac.il) , Nir Cohen (nirc@weizmann.ac.il)

Review Timeline:

Transfer from Review Commons:	24th Dec 22
Editorial Decision:	23rd Jan 23
Revision Received:	5th Mar 23
Editorial Decision:	8th Mar 23
Revision Received:	11th Mar 23
Accepted:	17th Mar 23

Editor: Stefanie Bohm/William Teale

Transaction Report: This manuscript was transferred to EMBO reports following peer review at Review Commons

Review COMMONS

(Note: With the exception of the correction of typographical or spelling errors that could be a source of ambiguity, letters and reports are not edited. Depending on transfer agreements, referee reports obtained elsewhere may or may not be included in this compilation. Referee reports are anonymous unless the Referee chooses to sign their reports.)

Review #1

1. Evidence, reproducibility and clarity:

Evidence, reproducibility and clarity (Required)

In this manuscript the authors develop an unbiased method to measure protein-protein interactions in cells through application of BirA biotin ligase technology. The novel feature of the approach is to append BirA to homologous ER translocon proteins (Sec61 and Ssh1) and then measure biotinylation of the AviTag when fused to the N-terminus of each protein in the yeast proteome. This is accomplished by mating the specific BirA tagged strain with a collection of 6000 yeast strains each with a distinct AviTagged protein. The level of AviTag biotinylation in diploid strains is measured by probing cell lysates applied to filters with fluorescent streptavidin. 2070 proteins displayed interactions with BirA-Sec61 and/or BirA-Ssh1 with a subset of secretory proteins showing preference for one of the translocons. The influence of ssh1 deletion on localization of the GFP-tagged protein library (4127 strains) was also determined and compared with protein interaction data. Moreover, analyses of signal peptides revealed that hydrophobicity of the N-terminal three residues can be sufficient to impart specificity for Sec61 or Ssh1 interaction. The reported findings support their primary conclusions. I have only a few suggestions to strengthen this study.

1. The Figure 1 legend indicates that the BirA tagged strains are mated with ~6000 AviTag yeast strains but results in Figure 2 pie chart account for 4812 total readouts. Presumably 1000 or more strains could not mate or did not produce viable diploids with the BirA tagged strains? It would be helpful to explain this differential.
2. If possible, suggest including more of the raw data (in supplementary) that supports the pie chart in Figure 2. Table S1 shows the 111 proteins that display preference for Ssh1 (out of 586 total interactors?) and the fold change (in rank order) for interaction preference. At a minimum, similar data on Sec61 preference and the list of positive interactors should be included. There may also be useful information in the relative biotinylation signal for each BirA and AviTag combination when significantly above background. This is presumably a readout of AviTag protein abundance, dwell time and orientation to BirA activity. The data could be useful to other investigators.
3. The authors might want to be more cautious in interpreting impact of the UPR on ssh1 phenotypes in the results and discussion. The Wilkinson et al 2002 paper referenced used very different conditions to detect UPR in ssh1 deletions strains. Jonikas et al 2009 does not detect a chronic UPR in ssh1 deletion cells and the conditions used in the current study seem more similar to the 2009 report. It seems more likely that deficits in translocating/localizing specific proteins causes the observed phenotypes instead of chronic UPR due to reduced ER levels of PDI.

2. Significance:

Significance (Required)

The reported findings support their primary conclusions. The technology development and results are significant, highly relevant and will be of interest to a broad readership in cell and membrane biology.

3. How much time do you estimate the authors will need to complete the suggested revisions:

Estimated time to Complete Revisions (Required)

(Decision Recommendation)

Less than 1 month

4. *Review Commons* values the work of reviewers and encourages them to get credit for their work. Select 'Yes' below to register your reviewing activity at Web of Science Reviewer Recognition Service (formerly Publons); note that the content of your review will not be visible on Web of Science.

Web of Science Reviewer Recognition

No

Review #2

1. Evidence, reproducibility and clarity:

Evidence, reproducibility and clarity (Required)

Summary:

Cohen et al. presented a high-throughput approach to analyze protein-(putative) substrate interactions in yeast using BirA biotin ligase and its acceptor peptide AVI tag. Using this system, the authors identified the common and unique substrates of translocation pores, Sec61 and Ssh1. Interestingly, the differential substrates between Sec61 and Ssh1 seem to be explained by the degree of hydrophobicity in signal peptide sequences, which was also nicely demonstrated by an experiment showing that swapping the first three amino acids of substrate proteins is sufficient to convert the substrate specificity. While I appreciate that the approach is high-throughput and simple (does not require mass spectrometers), there are some technical comments to be addressed.

1. Why was BirA used to study transient interactions? Biotinylation through BirA is slow (that is why it takes several hours to label proximity proteins) and thus it may not be suitable for capturing transient interactions. Instead, TurboID would be more suitable as the biotinylation reaction is faster than BirA. A reasonable explanation using BirA is required.
2. One key question is whether biotinylated proteins identified by this method are substrates or proteins just proximal to Sec61 or Ssh1 due to close cellular localization (e.g. ER membrane) or same protein complex members. An experiment or analysis would be required to confirm that the proteins they identified are indeed potential substrates.
3. Along the same line, if proteins identified by this approach are bona fide substrates of Sec61 and Ssh1, proteins having signal peptides should be enriched in the candidate list of substrates. However, it does not look like that according to Figure 2A where the secretome proteins/total proteins ratio appears to be similar among the 4 categories (e.g., Ssh1 preferring, No preference, and Not interacting or excluded). The authors should comment on this.
4. Figures 1-2: They should comment on the reproducibility of the method. How many independent experiments were performed? If performed, how was reproducibility of results?
5. Figure 3: It is important to know the overlap of proteins commonly identified in both the interaction screening and protein localization assay. A Venn diagram that compares results between the two high-throughput assays would be useful.
6. Figure 4A (GO term): The authors mentioned that " the most consistent and repeating GO term group was those related to budding and polarity process. These include: "Establishment or maintenance of cell polarity"; "Development process involved in reproduction"; "Bipolar cellular bud site selection"; "Cell budding" and "Structural constituent of cell wall". Are protein sets in these functional categories similar or different? I am asking because GO enrichment analysis often provides apparently different functional categories but similar protein sets are included.

Referees cross-commenting

The comments from reviewer #1 are reasonable and would further strengthen the quality of the paper.

2. Significance:

Significance (Required)

The approach is high-throughput and simple (does not require mass spectrometers).

The differential substrates between Sec61 and Ssh1 seem to be explained by the degree of hydrophobicity in signal peptide sequences, which was also nicely demonstrated by an experiment showing that swapping the first three amino acids of substrate proteins is sufficient to convert the substrate specificity.

3. How much time do you estimate the authors will need to complete the suggested revisions:

Estimated time to Complete Revisions (Required)

(Decision Recommendation)

Between 1 and 3 months

4. *Review Commons* values the work of reviewers and encourages them to get credit for their work. Select 'Yes' below to register your reviewing activity at Web of Science Reviewer Recognition Service (formerly Publons); note that the content of your review will not be visible on Web of Science.

Web of Science Reviewer Recognition

Yes

Point by point response to Reviewers' suggestions

Cohen et al, 2022

Reviewer #1

1. The Figure 1 legend indicates that the BirA tagged strains are mated with ~6000 AviTag yeast strains but results in Figure 2 pie chart account for 4812 total readouts. Presumably 1000 or more strains could not mate or did not produce viable diploids with the BirA tagged strains? It would be helpful to explain this differential.
We thank Reviewer #1 for pointing out this gap which occurred exactly as they have interpreted. We have now corrected the figure legend to say exactly how many strains were in the library (5330) and have clearly stated the attrition of strains.

2. If possible, suggest including more of the raw data (in supplementary) that supports the pie chart in Figure 2. Table S1 shows the 111 proteins that display preference for Ssh1 (out of 586 total interactors?) and the fold change (in rank order) for interaction preference. At a minimum, similar data on Sec61 preference and the list of positive interactors should be included. There may also be useful information in the relative biotinylation signal for each BirA and AviTag combination when significantly above background. This is presumably a readout of AviTag protein abundance, dwell time and orientation to BirA activity. The data could be useful to other investigators.
This is a very good suggestion. We have now added a supplementary table (Supplementary Table S2) with the interaction results for proteins that preferred Sec61 and proteins that did not show any preference.

3. The authors might want to be more cautious in interpreting impact of the UPR on ssh1 phenotypes in the results and discussion. The Wilkinson et al 2002 paper referenced used very different conditions to detect UPR in ssh1 deletions strains. Jonikas et al 2009 does not detect a chronic UPR in ssh1 deletion cells and the conditions used in the current study seem more similar to the 2009 report. It seems more likely that deficits in translocating/localizing specific proteins causes the observed phenotypes instead of chronic UPR due to reduced ER levels of PDI.
We agree that as result of the different conditions it is difficult to compare our data to the Wilkinson et al 2002 paper. We have therefore adjusted the text to remove this interpretation.

Reviewer #2

1. Why was BirA used to study transient interactions? Biotinylation through BirA is slow (that is why it takes several hours to label proximity proteins) and thus it may not be suitable for capturing transient interactions. Instead, TurboID would be more suitable as the biotinylation reaction is faster than BirA. A reasonable explanation using BirA is required.
We thank the reviewer for this comment which indeed also reflects our "process" of thinking. Originally, we did try to use TurboID to identify potential cargo proteins. We now have a very robust methodology to look at protein substrates by TurboID (see: https://www.biorxiv.org/content/10.1101/2022.04.27.489741v1) and so this would have obviously been the easier and faster method. However using this approach we mainly observed ribosome subunits and cytosolic proteins for Sec61 and very few, mostly cytosolic, proteins for Ssh1. Our interpretation of this is that since all biotinylation of TurboID strains

occurs in parallel there is “competition” from the highly abundant and strong interactors and this does not leave a possibility to detect the low-abundance and even more transient interactions that we would like to measure. It seems that although birA/AviTag are much slower, the specificity and singular ligation site that should be exposed also in co-translational-translocation events, are more suitable for this specific experimental setup. We have now explained this also in the text.

2. One key question is whether biotinylated proteins identified by this method are substrates or proteins just proximal to Sec61 or Ssh1 due to close cellular localization (e.g. ER membrane) or same protein complex members. An experiment or analysis would be required to confirm that the proteins they identified are indeed potential substrates.
This is indeed an extremely important point and we have now carefully addressed it in the text. We are certain that the reviewer is right and that many of the biotinylated proteins are same complex members and cytosolic components that happen to be in proximity (maybe regulators?) just as the reviewer suggested. We now clearly write this in the results section. This is why we focused on signal peptide containing proteins. These proteins CAN NOT be complex members nor biotinylated simply due to proximal location on the ER membrane. This is since they reside inside the lumen of the ER if they are soluble or are inserted (if they contain also a transmembrane domain) with their tagged N’ facing the lumen of the ER (So called Type I proteins). The only way such proteins could be biotinylated by the slow BirA on the cytosolic surface is if they passed through the pore of the translocon.

3. Along the same line, if proteins identified by this approach are bona fide substrates of Sec61 and Ssh1, proteins having signal peptides should be enriched in the candidate list of substrates. However, it does not look like that according to Figure 2A where the secretome proteins/total proteins ratio appears to be similar among the 4 categories (e.g., Ssh1 preferring, No preference, and Not interacting or excluded). The authors should comment on this.
We thank Reviewer #2 for highlighting this point that was not clear from our text and figures. There is definitely an enrichment of Signal Peptide (SP) containing proteins amongst the proteins that we think are bona fide substrates however this was not visualized clearly. To highlight this point we have modified Figure 2 and added a bar graph showing the distribution of SP and TMD proteins within the potential secretome. This graph now highlights the enrichment of SP containing proteins in the groups of proteins that preferred Sec61 or Ssh1 in comparison to the group that did not show a preference.
We also now add a citation from a new manuscript from the Hegde lab that suggests that indeed soluble SP containing proteins are the key clients for the translocon pore (https://pubmed.ncbi.nlm.nih.gov/36261528/). We have also added a section to the discussion as to why we do not see differential enrichment of SRP or its receptor on either pore although in the past this was suggested to be the key difference between the two translocons.

4. Figures 1-2: They should comment on the reproducibility of the method. How many independent experiments were performed? If performed, how was reproducibility of results?

Thank you for highlighting that this was not clarified enough – we have now extended the materials and methods section to make all of the above issues clear and apparent. In short, we performed 3 <u>biological</u> repetitions for each experiment and for each biological repeat we performed 3 <u>technical</u> repeats making our results altogether rely on 9 repeats. We then excluded proteins in two cases

1. *If strains were missing in either of the collections (so there was no complete set to compare them) – this caused us to drop 661 strains.*
2. *In cases where the proteins were expressed at very low levels of extracted poorly in our assay – we defined this as the signal being ten standard deviations (or more) lower than the rest of the signals on the same membrane – this caused us to lose an additional 93 strains.*

Importantly, the SD between all 9 repeats never rose above 3 (see graph below showing al strains arranged by order in library and the SD between all 9 repeats) and also now stated clearly in the text) hence we think that our method is highly reproducible

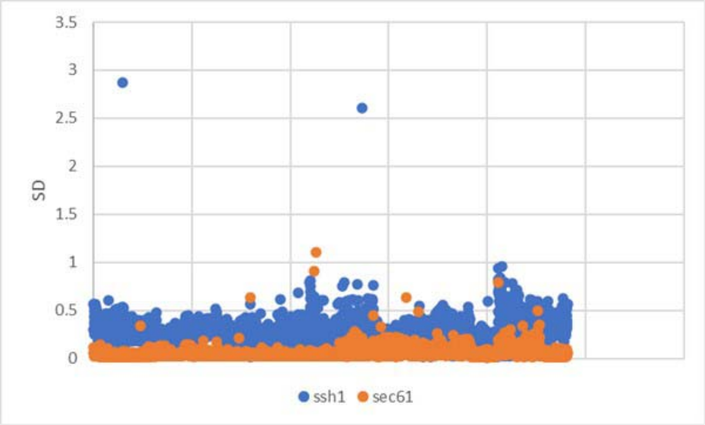

5. Figure 3: It is important to know the overlap of proteins commonly identified in both the interaction screening and protein localization assay. A Venn diagram that compares results between the two high-throughput assays would be useful.
We have indeed considered making this Venn diagram (See below). However, since the connection between the screens is not direct due to the fact that the protein localization is downstream to translocation events or maybe completely independent of it, we found that the number of specific proteins that are in both screens is low. However, there is a much larger overlap in joining processes and functions, therefore we decide to make the grouping showed in Figure 4B. We would prefer not to show this figure in the manuscript however we leave this to editorial decision.

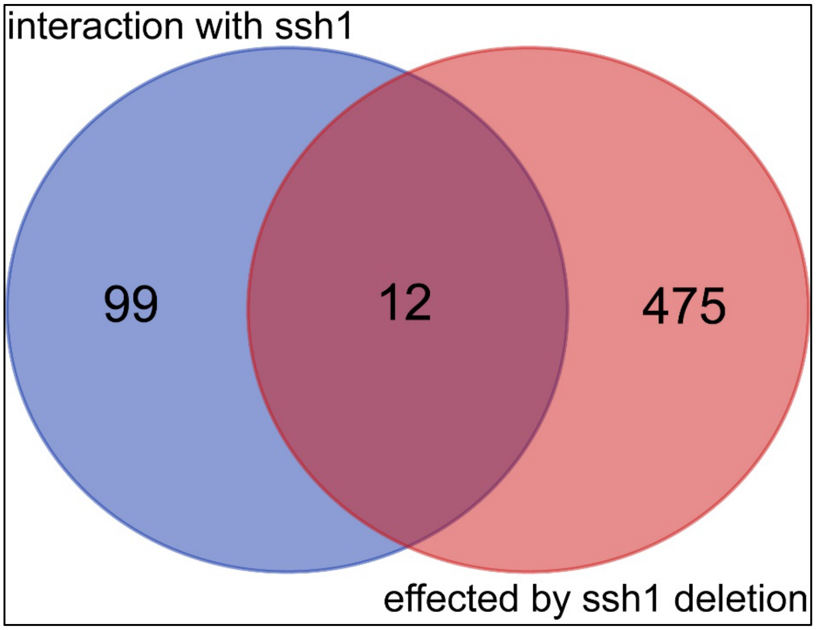

6. Figure 4A (GO term): The authors mentioned that " the most consistent and repeating GO term group was those related to budding and polarity process. These include: "Establishment or maintenance of cell polarity"; "Development process involved in reproduction"; "Bipolar cellular bud site selection"; "Cell budding" and "Structural constituent of cell wall". Are protein sets in these functional categories similar or different? I am asking because GO enrichment analysis often provides apparently different functional categories but similar protein sets are included.

Indeed, this reviewer is totally correct and this is also the case here to some extent. There is some level of overlap between the GO terms. However, in our case this overlap is quite small: Out of the 77 genes that have one of those GO terms assigned only 2 have all 4, 9 have 3 and 4 have 2 of the GO terms therefore we believe that in this case this issue with GO terms hierarchy and assignment is not redundant. We are happy to highlight this in the figure or text if this is deemed to be important.

1st Editorial Decision 23rd Jan 2023

Dear Dr. Schuldiner, dear Maya,

Thank you for submitting your revised Review Commons manuscript to The EMBO Journal (EMBOJ-2023-113385). We sent it back to the two initial referees and have now received their reports (please see below). Referee #1 has a remaining comment, which you should address, but I am happy to say that we can overall proceed towards publication in The EMBO Journal. Therefore, I would ask you to please also resolve a number of editorial issues that are listed in detail below. Please use the document that the data editors have added their comments to for any changes, both those to address the referee comment, as well as those to resolve the editorial issues (see below). If you have any further questions regarding the specific points listed below or the final manuscript version, please feel free to contact me.

Thank you again for giving us the chance to consider your manuscript for The EMBO Journal.

Kind regards,

Stefanie

Stefanie Boehm
Editor
The EMBO Journal

Formatting changes required for revised version of the manuscript

1) Please provide a synopsis image and text:
a) Three to four 'bullet points' highlighting the main findings of your study
b) A short 'blurb' text summarizing in two sentences the study (max. 250 characters). Bullet points and standfirst text should be submitted as a separate manuscript file in LaTeX, RTF or MS Word format.
c) A "synopsis image" (Illustrator, PowerPoint, OmniGraffle or jpeg format), which can be used as "visual title" for the synopsis section of your paper (image dimensions should be 550 (w) x 300-600 (h) px).
For further information please refer to: https://www.embopress.org/page/journal/14602075/authorguide#manuscriptpreparation

2) Please update the Conflict of Interest statement to "Disclosure and Competing Interests Statement" and explicitly state if there is nothing to declare.

3) Please provide up to five keywords, which may or may not appear in the title, should be given in alphabetical order, below the abstract, each separated by a slash (/).

4) Please remove the "Author contributions" section from the manuscript. CRediT has replaced the traditional author contributions section because it offers a systematic machine readable author contributions format that allows for more effective research assessment. Please remove this information from the manuscript and use the free text boxes beneath each contributing author's name in our system to add specific details on the author's contribution. More information is available in our guide to authors.

5) Please update the references to the journal's format and remove the DOI numbers.

6) Please add a reference to figure 4D. All figures and their individual panels must be mentioned in the main text.

7) Please complete and upload the author checklist.

8) Please add the Data Availability section and ensure all datasets are accessible.

9) Please upload all main and EV figures as individual image files using the file type "Figure File" (all without the figure legends, these should be included in the main manuscript).

10) Please change the nomenclature of Table S1-S4 to Dataset EV1- EV4. Please include a respective legend in each file, for example as a separate sheet in the Excel workbook. Please also remember to update all references to this data in the text.

11) Please update the order of the sections in the manuscript to the journal's standard. Please also see:
https://www.embopress.org/page/journal/14602075/authorguide#researcharticleguide

12) Our data editors have raised several queries with the data descriptors in the figure legends, which you will find as comments

in the Word document EMBOJ-2022-113385_figure_QC.docx (as part of your submission and attached to this message). I would appreciate if you incorporated the requested final text modifications and answered the figure legend queries directly in this version, uploading the edited main text document upon resubmission with changes/additions still highlighted via the "Track changes" option.

Further information is available in our Guide For Authors:
https://www.embopress.org/page/journal/14602075/authorguide

****

Revision to The EMBO Journal should be submitted online within 90 days, unless an extension has been requested and approved by the editor; please click on the link below to submit the revision online before 23rd Apr 2023:

Link Not Available
****

Referee #1:

The authors have addressed most of my concerns. Regarding the comments #4 about the reproducibility of the assay, they should show the result in main or supplementary figure as quality of the dataset is important for such large-scale assays.

Referee #2:

This revised manuscript fully addresses my initial comments and I enthusiastically support publication of this work. The technology development and new findings are significant and will be of interest to a broad readership.

Dear Stefanie,

We are really excited about the opportunity to publish with you and have made sure to address all of the reviewers and editorial points that you mentioned below.

I have now uploaded all of the new materials and hope that this is satisfactory. Please let me know if you require anything else.

all best,
Maya

1st Revision - Editorial Decision **8th Mar 2023**

Dear Maya,

We have now received re-review reports from the two referees that initially appraised your manuscript for Review Commons. As you will see, you have addressed their concerns satisfactorily. Before I can finally accept the manuscript though, I have noticed some small editorial points that remain and need to be addressed. In this regard would you please:

- state how the relevant error bars were calculated in the legend to figure 5A,
- replace the second sentence in the figure legend for 5B to the suggested "Shown are Pdi1 and Mpd2 (two PDI family members that showed an interaction preference to Ssh1) and Mpd1 (a protein that showed an interaction preference for Sec61)",
- consider the following suggestions for the fifth sentence of the introduction "Hence, there is a general need in cell biology for approaches able to describe the particular range of any given protein's functions in vivo, even when that protein belongs to a large and closely-related family.",
- provide the synopsis image as a jpeg, tiff or a png file,
- provide page numbers for the Table of Contents and use the nomenclature 'Appendix Figure S1' and so on; please also remove supplementary figure legends from the manuscript file and add them below each figure in the Appendix PDF, and
- add the following sentence to the Disclosure and Competing Interests Statement "Maya Schuldiner is an EBM Member. This has no bearing on the editorial consideration of this article for publication."

Best wishes,

William

Use the link below to submit your revision:

https://emboj.msubmit.net/cgi-bin/main.plex

Dear William

I have just finished uploading all of the required materials. Specifically please see below each of your points what has been done. I hope all is now in order and thank you for being so careful!
All best,
Maya

Dear Maya,

We have now received re-review reports from the two referees that initially appraised your manuscript for Review Commons. As you will see, you have addressed their concerns satisfactorily. Before I can finally accept the manuscript though, I have noticed some small editorial points that remain and need to be addressed. In this regard would you please:

- state how the relevant error bars were calculated in the legend to figure 5A,
This has now been added to the text and a new article file has been uploaded to the system

- replace the second sentence in the figure legend for 5B to the suggested "Shown are Pdi1 and Mpd2 (two PDI family members that showed an interaction preference to Ssh1) and Mpd1 (a protein that showed an interaction preference for Sec61)",
Done and in new article file as above

- consider the following suggestions for the fifth sentence of the introduction "Hence, there is a general need in cell biology for approaches able to describe the particular range of any given protein's functions in vivo, even when that protein belongs to a large and closely-related family.",
Agreed - this is a great way of phrasing it. Now in the new text file.

- provide the synopsis image as a jpeg, tiff or a png file,
Now provided as a .jpg

- provide page numbers for the Table of Contents and use the nomenclature 'Appendix Figure S1' and so on; please also remove supplementary figure legends from the manuscript file and add them below each figure in the Appendix PDF, and
I hope we did this right - if there is a problem please let us know. since there was only one sup Figure the appendix was a bit small.

- add the following sentence to the Disclosure and Competing Interests Statement "Maya Schuldiner is an EBM Member. This has no bearing on the editorial consideration of this article for publication."
Added in the new text file

Many thanks for all of your help
Maya

Dear Maya,

I am pleased to inform you that your manuscript has been accepted for publication in the EMBO Journal.

Congratulations on pulling off this really ambitious project so well!

Please note that it is EMBO Journal policy for the transcript of the editorial process (containing referee reports and your response letter) to be published as an online supplement to each paper. If you do NOT want this, you will need to inform the Editorial Office via email immediately. More information is available here: https://www.embopress.org/page/journal/14602075/authorguide#transparentprocess

Your manuscript will be processed for publication in the journal by EMBO Press. Manuscripts in the PDF and electronic editions of The EMBO Journal will be copy edited, and you will be provided with page proofs prior to publication. Please note that supplementary information is not included in the proofs.

You will be contacted by Wiley Author Services to complete licensing and payment information. The required 'Page Charges Authorization Form' is available here: https://www.embopress.org/pb-assets/embo-site/tej_apc.pdf - please download and complete the form and return to embopressproduction@wiley.com

EMBO Press participates in many Publish and Read agreements that allow authors to publish Open Access with reduced/no publication charges. Check your eligibility: https://authorservices.wiley.com/author-resources/Journal-Authors/open-access/affiliation-policies-payments/index.html

Should you be planning a Press Release on your article, please get in contact with embojournal@wiley.com as early as possible, in order to coordinate publication and release dates.

If you have any questions, please do not hesitate to call or email the Editorial Office. Thank you for your contribution to The EMBO Journal.

Yours sincerely,

William

William Teale, PhD
Editor
The EMBO Journal
w.teale@embojournal.org

** Click here to be directed to your login page: https://emboj.msubmit.net

EMBO Press Author Checklist

Corresponding Author Name: Schuldiner Maya	
Journal Submitted to: EMBO J	
Manuscript Number: EMBOJ-2022-113385	

USEFUL LINKS FOR COMPLETING THIS FORM
The EMBO Journal - Author Guidelines
EMBO Reports - Author Guidelines
Molecular Systems Biology - Author Guidelines
EMBO Molecular Medicine - Author Guidelines

Reporting Checklist for Life Science Articles (updated January 2022)
This checklist is adapted from Materials Design Analysis Reporting (MDAR) Checklist for Authors. MDAR establishes a minimum set of requirements in transparent reporting in the life sciences (see Statement of Task: 10.31222/osf.io/9sm4x). Please follow the journal's guidelines in preparing your manuscript.
Please note that a copy of this checklist will be published alongside your article.

Abridged guidelines for figures
1. Data
The data shown in figures should satisfy the following conditions:
- the data were obtained and processed according to the field's best practice and are presented to reflect the results of the experiments in an accurate and unbiased manner.
- ideally, figure panels should include only measurements that are directly comparable to each other and obtained with the same assay.
- plots include clearly labeled error bars for independent experiments and sample sizes. Unless justified, error bars should not be shown for technical replicates.
- if n<5, the individual data points from each experiment should be plotted. Any statistical test employed should be justified.
- Source Data should be included to report the data underlying figures according to the guidelines set out in the authorship guidelines on Data Presentation.

2. Captions
Each figure caption should contain the following information, for each panel where they are relevant:
- a specification of the experimental system investigated (eg cell line, species name).
- the assay(s) and method(s) used to carry out the reported observations and measurements.
- an explicit mention of the biological and chemical entity(ies) that are being measured.
- an explicit mention of the biological and chemical entity(ies) that are altered/varied/perturbed in a controlled manner.
- the exact sample size (n) for each experimental group/condition, given as a number, not a range;
- a description of the sample collection allowing the reader to understand whether the samples represent technical or biological replicates (including how many animals, litters, cultures, etc.).
- a statement of how many times the experiment shown was independently replicated in the laboratory.
- definitions of statistical methods and measures:
  - common tests, such as t-test (please specify whether paired vs. unpaired), simple χ2 tests, Wilcoxon and Mann-Whitney tests, can be unambiguously identified by name only, but more complex techniques should be described in the methods section;
  - are tests one-sided or two-sided?
  - are there adjustments for multiple comparisons?
  - exact statistical test results, e.g., P values = x but not P values < x;
  - definition of 'center values' as median or average;
  - definition of error bars as s.d. or s.e.m.

Please complete ALL of the questions below.
Select "Not Applicable" only when the requested information is not relevant for your study.

Materials

Newly Created Materials	Information included in the manuscript?	In which section is the information available? (Reagents and Tools Table, Materials and Methods, Figures, Data Availability Section)
New materials and reagents need to be available; do any restrictions apply?	Not Applicable	

Antibodies	Information included in the manuscript?	In which section is the information available? (Reagents and Tools Table, Materials and Methods, Figures, Data Availability Section)
For **antibodies** provide the following information:<br>- Commercial antibodies: RRID (if possible) or supplier name, catalogue number and or/clone number<br>- Non-commercial: RRID or citation	Yes	Materials and Methods

DNA and RNA sequences	Information included in the manuscript?	In which section is the information available? (Reagents and Tools Table, Materials and Methods, Figures, Data Availability Section)
**Short novel DNA or RNA including primers, probes**: provide the sequences.	Yes	Table EV4

Cell materials	Information included in the manuscript?	In which section is the information available? (Reagents and Tools Table, Materials and Methods, Figures, Data Availability Section)
**Cell lines:** Provide species information, strain. Provide accession number in repository **OR** supplier name, catalog number, clone number, and/**OR** RRID.	Not Applicable	
**Primary cultures:** Provide species, strain, sex of origin, genetic modification status.	Not Applicable	
Report if the cell lines were recently **authenticated** (e.g., by STR profiling) and tested for mycoplasma contamination.	Not Applicable	

Experimental animals	Information included in the manuscript?	In which section is the information available? (Reagents and Tools Table, Materials and Methods, Figures, Data Availability Section)
**Laboratory animals or Model organisms:** Provide species, strain, sex, age, genetic modification status. Provide accession number in repository **OR** supplier name, catalog number, clone number, **OR** RRID.	Not Applicable	
**Animal observed in or captured from the field:** Provide species, sex, and age where possible.	Not Applicable	
Please detail **housing and husbandry conditions**.	Not Applicable	

Plants and microbes	Information included in the manuscript?	In which section is the information available? (Reagents and Tools Table, Materials and Methods, Figures, Data Availability Section)
**Plants:** provide species and strain, ecotype and cultivar where relevant, unique accession number if available, and source (including location for collected wild specimens).	Not Applicable	
**Microbes:** provide species and strain, unique accession number if available, and source.	Yes	Materials and Methods and Table EV4

Human research participants	Information included in the manuscript?	In which section is the information available? (Reagents and Tools Table, Materials and Methods, Figures, Data Availability Section)
If collected and within the bounds of privacy constraints report on age, sex and gender or ethnicity for all study participants.	Not Applicable	

Core facilities	Information included in the manuscript?	In which section is the information available? (Reagents and Tools Table, Materials and Methods, Figures, Data Availability Section)
If your work benefited from core facilities, was their service mentioned in the acknowledgments section?	Yes	

Design

Study protocol	Information included in the manuscript?	In which section is the information available? (Reagents and Tools Table, Materials and Methods, Figures, Data Availability Section)
If study protocol has been **pre-registered, provide DOI in the manuscript**. For clinical trials, provide the trial registration number **OR** cite DOI.	Not Applicable	
Report the **clinical trial registration number** (at ClinicalTrials.gov or equivalent), where applicable.	Not Applicable	

Laboratory protocol	Information included in the manuscript?	In which section is the information available? (Reagents and Tools Table, Materials and Methods, Figures, Data Availability Section)
Provide DOI OR other citation details if **external detailed step-by-step protocols** are available.	Not Applicable	

Experimental study design and statistics	Information included in the manuscript?	In which section is the information available? (Reagents and Tools Table, Materials and Methods, Figures, Data Availability Section)
Include a statement about **sample size** estimate even if no statistical methods were used.	Yes	Materials and Methods
Were any steps taken to minimize the effects of subjective bias when allocating animals/samples to treatment (e.g. **randomization procedure**)? If yes, have they been described?	Not Applicable	
Include a statement about **blinding** even if no blinding was done.	Not Applicable	
Describe **inclusion/exclusion criteria** if samples or animals were excluded from the analysis. Were the criteria pre-established?<br><br>If sample or data points were omitted from analysis, report if this was due to attrition or intentional exclusion and provide justification.	Yes	Materials and Methods
For every figure, are **statistical tests** justified as appropriate? Do the data meet the assumptions of the tests (e.g., normal distribution)? Describe any methods used to assess it. Is there an estimate of variation within each group of data? Is the variance similar between the groups that are being statistically compared?	Not Applicable	

Sample definition and in-laboratory replication	Information included in the manuscript?	In which section is the information available? (Reagents and Tools Table, Materials and Methods, Figures, Data Availability Section)
In the figure legends: state number of times the experiment was **replicated** in laboratory.	Not Applicable	
In the figure legends: define whether data describe **technical or biological replicates**.	Not Applicable	

Ethics

Ethics	Information included in the manuscript?	In which section is the information available? (Reagents and Tools Table, Materials and Methods, Figures, Data Availability Section)
Studies involving **human participants**: State details of **authority granting ethics approval** (IRB or equivalent committee(s), provide reference number for approval.	Not Applicable	
Studies involving **human participants**: Include a statement confirming that **informed consent** was obtained from all subjects and that the experiments conformed to the principles set out in the WMA Declaration of Helsinki and the Department of Health and Human Services Belmont Report.	Not Applicable	
Studies involving **human participants:** For publication of **patient photos**, include a statement confirming that consent to publish was obtained.	Not Applicable	
Studies involving experimental **animals**: State details of **authority granting ethics approva**l (IRB or equivalent committee(s), provide reference number for approval. Include a statement of compliance with ethical regulations.	Not Applicable	
Studies involving **specimen and field samples:** State if relevant **permits** obtained, provide details of authority approving study; if none were required, explain why.	Not Applicable	

Dual Use Research of Concern (DURC)	Information included in the manuscript?	In which section is the information available? (Reagents and Tools Table, Materials and Methods, Figures, Data Availability Section)
Could your study fall under dual use research restrictions? Please check biosecurity documents and list of **select agents and toxins** (CDC): https://www.selectagents.gov/sat/list.htm	Not Applicable	
If you used a select agent, is the security level of the lab appropriate and reported in the manuscript?	Not Applicable	
If a study is subject to dual use research of concern regulations, is the name of the **authority granting approval and reference number** for the regulatory approval provided in the manuscript?	Not Applicable	

Reporting

The MDAR framework recommends adoption of discipline-specific guidelines, established and endorsed through community initiatives. Journals have their own policy about requiring specific guidelines and recommendations to complement MDAR.

Adherence to community standards	Information included in the manuscript?	In which section is the information available? (Reagents and Tools Table, Materials and Methods, Figures, Data Availability Section)
State if relevant guidelines or checklists (e.g., **ICMJE, MIBBI, ARRIVE, PRISMA**) have been followed or provided.	Not Applicable	
For **tumor marker prognostic studies,** we recommend that you follow the **REMARK** reporting guidelines (see link list at top right). See author guidelines, under 'Reporting Guidelines'. Please confirm you have followed these guidelines.	Not Applicable	
For **phase II and III randomized controlled trials**, please refer to the **CONSORT** flow diagram (see link list at top right) and submit the CONSORT checklist (see link list at top right) with your submission. See author guidelines, under 'Reporting Guidelines'. Please confirm you have submitted this list.	Not Applicable	

Data Availability

Data availability	Information included in the manuscript?	In which section is the information available? (Reagents and Tools Table, Materials and Methods, Figures, Data Availability Section)
Have **primary datasets** been deposited according to the journal's guidelines (see 'Data Deposition' section) and the respective accession numbers provided in the Data Availability Section?	Yes	Data availability section
Were **human clinical and genomic datasets** deposited in a public access-controlled repository in accordance to ethical obligations to the patients and to the applicable consent agreement?	Not Applicable	
Are **computational models** that are central and integral to a study available without restrictions in a machine-readable form? Were the relevant accession numbers or links provided?	Not Applicable	
If publicly available data were reused, provide the respective **data citations in the reference list**.	Not Applicable	
